# Jejunal Gastrointestinal Stromal Tumor Presenting as Hemorrhagic Shock

**DOI:** 10.7759/cureus.62155

**Published:** 2024-06-11

**Authors:** Manish Wadhwa, Navroop Nagra, Neha Singh, Sahil Kumar, Endashaw Omer

**Affiliations:** 1 Internal Medicine, North Alabama Medical Center, Florence, USA; 2 Gastroenterology, University of Louisville, Louisville, USA; 3 College of Medicine, Jinnah Sindh Medical University, Karachi, PAK

**Keywords:** small intestinal tumors, hemorrhagic shock, acute gi bleed, jejunal, gastrointestinal tumor (gist)

## Abstract

Gastrointestinal stromal tumors (GISTs) are rare tumors of the gastrointestinal (GI) tract. Intermittent GI bleeding is the most common manifestation. Massive GI bleeding leading to syncopal episodes and hemorrhagic shock is a rare presentation of these tumors. Herein, we describe a case of a jejunal GIST presenting as massive bleeding.

## Introduction

Gastrointestinal stromal tumor (GIST) accounts for less than 1% of all tumors within the gastrointestinal (GI) tract [1]. They arise from the interstitial cells of Cajal, also known as GI pacemaker cells, which regulate peristalsis by acting as an interface between the GI autonomic nervous system of the bowel wall and smooth muscle layer, and can occur anywhere in the GI tract, the most common site being the stomach, followed by the small intestine, large intestine, and esophagus. Most cases occur in patients over 50 years of age with a mean age of around 64 [1-3]. Usually, GIST presents as intermittent GI bleeding (42% of cases), and massive life-threatening bleeding requiring urgent intervention is a very rare presentation [1]. Due to the difficulty in accessing the small intestine, diagnosing small bowel bleeding by conventional endoscopy becomes a challenging task [4]. This case report describes a jejunal GIST manifesting as repeated syncopal episodes with bright red blood per rectum and hemorrhagic shock.

## Case presentation

A 66-year-old man with type 2 diabetes mellitus (DM) and hypertension presented to the hospital with complaints of dizziness and fatigue for two days. Vital signs were stable on presentation. Initial labs were unremarkable except for hemoglobin of 7.2 g/dL. The patient had a bowel movement full of bright red blood while in the ER. He denied any prior history of blood in the stool. He underwent esophagogastroduodenoscopy (EGD) and colonoscopy the next day. EGD was normal, and colonoscopy showed blood-stained colonic mucosa in its entirety along with moderate diffuse diverticulosis. A few hours after the colonoscopy, he had a large bowel movement with fresh red blood, which led to hemodynamic instability and syncopal episode, and he was transferred to the ICU on pressors. CT angiogram (CTA) showed contrast extravasation in the loop of small bowel in the right mid-abdomen at the level of umbilicus consistent with active hemorrhage. He continued to have bloody bowel movements requiring multiple units of blood along with vasopressor support. Interventional radiology (IR) did an angiogram, which revealed no active bleeding. General surgery was consulted, considering the amount of bleeding, and after discussion with the surgery team, the GI team performed a repeat colonoscopy in the operating room.

**Figure 1:**
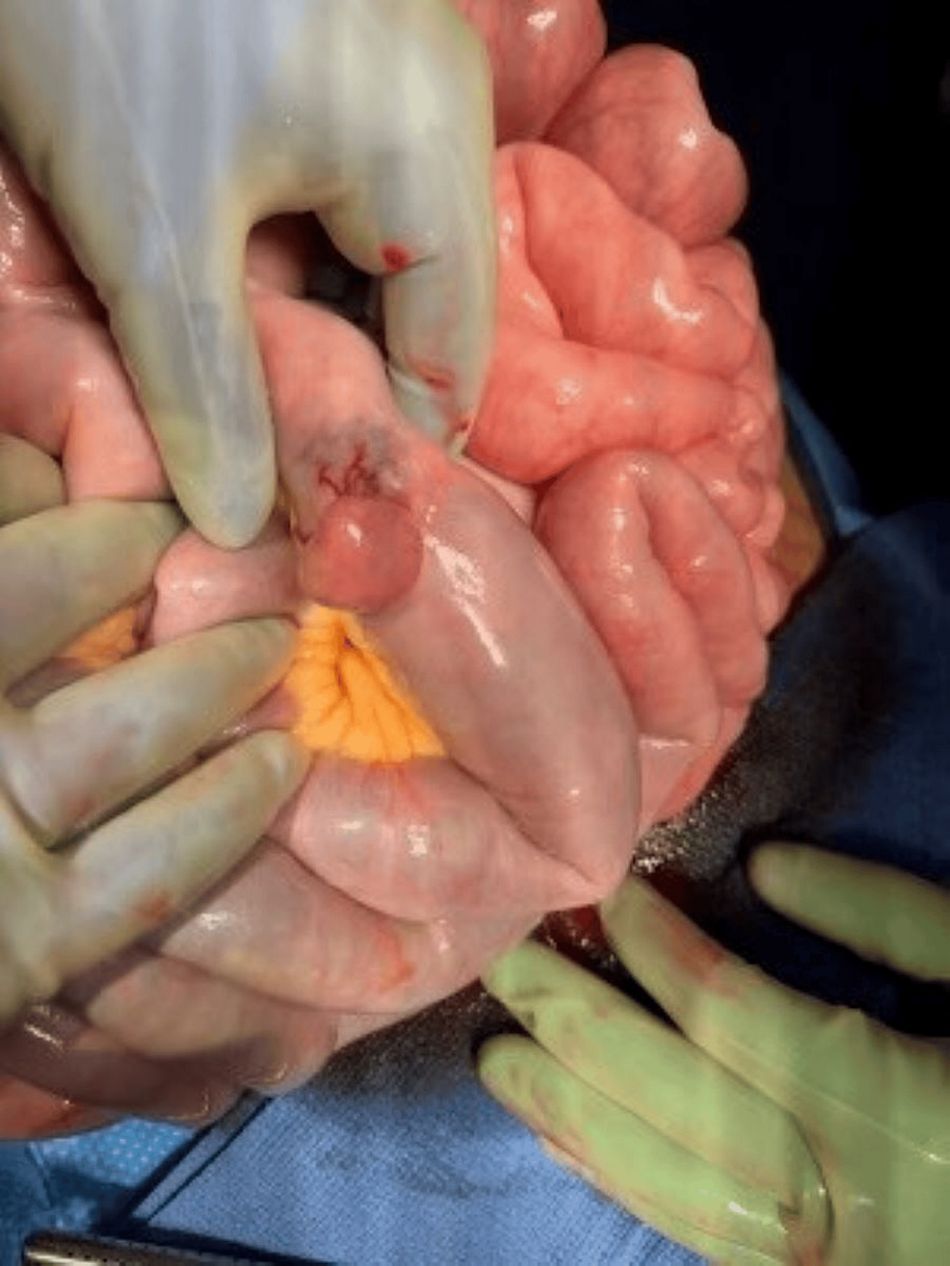
Intraoperative jejunal tumor

**Figure 2:**
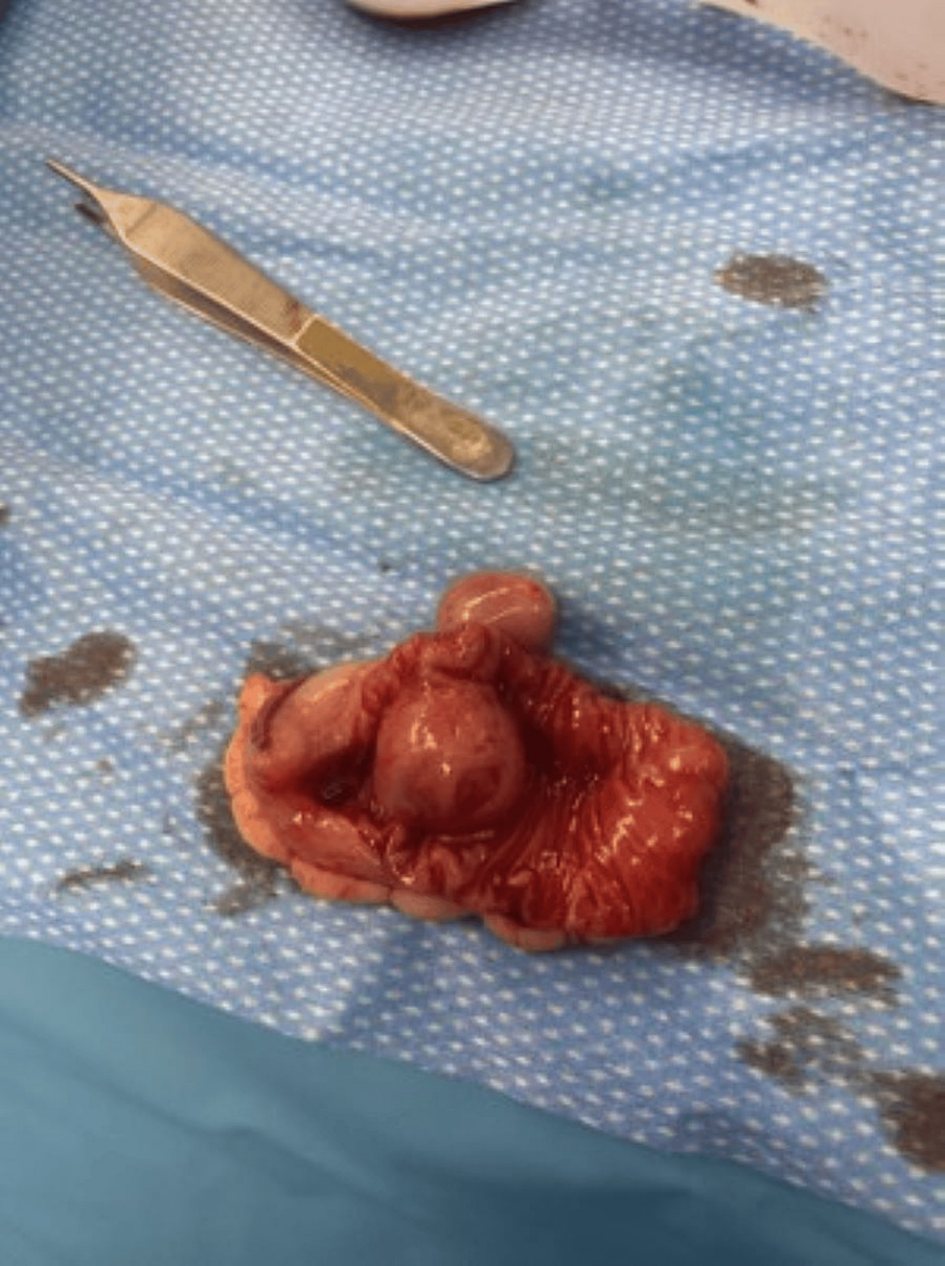
Resected jejunal segment with the tumor

The colonoscopy showed red blood in the distal ileum and entire colon, and no active bleeding was seen. Intraoperative push enteroscopy and open laparotomy were performed after the colonoscopy, which did not show any bleeding in the stomach, duodenum, or proximal jejunum. The general surgery team advanced the enteroscope manually, deep into the mid jejunum with no bleeding seen in the small bowel lumen. During the examination of the small bowel via laparotomy, a 2-3 cm serosal lesion was found in the distal jejunum with a clear transition point of color change (Figure 1). The small bowel distal to the lesion was full of blood and appeared maroon-colored, and a small bowel proximal to the lesion was normal pink color from the serosal side. The lesion was resected, and the pathology showed a 3 x 2.5 x 2.5 cm mass with an 0.7 cm ulcerated area through the mucosa (Figure 2). Histological findings were suggestive of a grade 1 spindle cell subtype GIST with a mitotic index of <2/50 HPF with negative resection margins. Genetic studies showed an exon 11 Kit gene mutation. Immunohistochemical studies were positive for KIT (CD 117), DOG 1, CD 34, and negative for desmin and S-100. Postoperatively, the patient remained stable without any further episodes of hematochezia. The patient tolerated feeding well and was discharged with outpatient follow-up with surgery and oncology. 

## Discussion

GIST can occur anywhere in the GI tract; the stomach accounts for 50-60% of cases, the small intestine for 20-30%, the colon or rectum for 5-10 %, the esophagus for <5 %, and the peritoneum and mesentery for <1 % of cases. However, small intestinal GISTs account for only 8.4% of small intestinal malignancies. [5]. It is a class of subepithelial lesion (SEL) of the GI tract. 

The clinical symptoms associated with small bowel GIST can vary from abdominal pain, anemia, bleeding, and distension to the acute abdomen with peritonitis [6]. Diagnosing small intestine GISTs can be an arduous task as the small intestine is sometimes inaccessible by conventional endoscopy. Patients presenting with GI bleeding symptoms with normal upper and lower endoscopy findings should raise a suspicion of small intestinal bleeding. GIST arising from the jejunum is one of the least common causes of massive small intestinal bleeding.

According to the latest National Comprehensive Cancer Network guidelines, a CT scan of the abdomen is the first-line investigation not only for the evaluation and staging but also for the monitoring of the treatment response. GIST on contrast-enhanced CT shows the characteristic of a well-defined soft tissue of relatively low density, which is homogenous [7]. Although CT is an essential test for the diagnosis of GIST, GI endoscopy, both upper and lower, helps to exclude other potential causes of patient symptoms even if they cannot detect lesions [8]. Moreover, adding endoscopic ultrasound helps to obtain an endoscopic biopsy to confirm the disease in addition to providing a wide range of view, especially if it is related to the gastric or duodenal walls [8,9].

Histologically, GISTs are of various morphologic types, including the spindle-shaped cell type (70%), epithelial cell type (20%), and mixed type (10%). Two other mesenchymal tumors are leiomyomas and neuromas, which can be differentiated only with immunostaining and not by conventional immunostaining. Ninety percent (90%) of GISTs have activating mutations in the proto-oncogenes that code for the receptor tyrosine kinases KIT (75-80%) or platelet-derived growth factor (PDGFRA). 

On IHC, 95% of lesions are positive for CD 117 (KIT), making it the most prominent diagnostic marker for GIST [10]. It is worth mentioning that not all KIT-positive GIST lesions are positive for its mutations. These tumors will stain positive for KIT with no detectable mutations on the gene itself. These patients do not show a good response to imatinib therapy [11].

## Conclusions

Small intestinal bleeding is not a very common presentation of GISTs arising from the jejunum. Due to the small intestine inaccessibility by conventional endoscopy, the diagnosis of small intestine GISTs becomes an arduous task. Normal findings from an upper and lower endoscopy in a patient presenting with GI bleeding symptoms should raise a suspicion of small intestinal bleeding. Complete surgical excision is the mainstay of management for small intestine GISTs.
